# A Forme Fruste of Marfan Syndrome: A Case Report

**DOI:** 10.7759/cureus.31231

**Published:** 2022-11-08

**Authors:** Nejood Alsheikh, Samira A Hawsawi, Abeer AlGhamdi, Lolo Alkhwaiter, Aseel Alsulimani, Ihab Suliman

**Affiliations:** 1 Medicine and Surgery, College of Medicine, King Saud Bin Abdulaziz University for Health Sciences, Riyadh, SAU; 2 Medicine and Surgery, King Saud Bin Abdulaziz University for Health Sciences, Riyadh, SAU; 3 Cardiology, King Abdulaziz Medical City, King Abdulaziz Cardiac Center, Ministry of National Guard Health Affairs, Riyadh, SAU

**Keywords:** arachnodactyly, pneumothorax, fibrillin-1, connective tissue disorder, marfan syndrome

## Abstract

Marfan syndrome (MFS), an inherited connective tissue disorder, is caused by a mutation in the FBN1 gene. MFS is characterized by manifestations in the musculoskeletal system (joint laxity, scoliosis), the cardiovascular system (aortic dilation), and the ocular system (ectopic lens). We report a case of a 37-year-old male with a genetically confirmed MFS. His mother and brother were also both confirmed cases of MFS. While the patient exhibited the characteristic physical features of MFS in general appearance, he did not show any cardiac manifestations of the disease. This report highlights a case of the familial occurrence of MFS and emphasizes the importance of recognizing the forme fruste of MFS.

## Introduction

Marfan syndrome (MFS) is an autosomal-dominant disorder affecting the connective tissue. It is caused by mutations in the recessive fibrillin 1 (FBN1) gene. It has an estimated prevalence of one in 3000-5000 individuals [1]. The disease affects both genders and all races equally. Moreover, in almost half of the individuals with MFS, there was a reported positive family history, whereas about 25-30% of the patients had a negative family history [2].

The cardinal manifestations of the syndrome are ocular findings such as myopia. Musculoskeletal manifestations of MFS include joint laxity and bone overgrowth, leading to chest deformity and scoliosis. The cardiovascular system manifestations are the main cause of morbidity and early mortality in MFS. They include dilatation of the aorta, which predisposes MFS patients to recurrent aortic tears, rupture, and dissections. The prophylactic medical treatment to protect the aorta includes regular follow-ups to help prevent or delay serious complications [2]. However, even after they undergo Bentall surgery, these patients are still at a high risk of complications and are prone to infective endocarditis [2,3]. Furthermore, they are at risk of other cardiac complications including prolapse of the mitral valve with or without regurgitation, tricuspid valve prolapse, and proximal pulmonary artery enlargement. Prolonged and severe mitral or/and aortic valve regurgitation leads to a higher risk of left ventricular dysfunction and, in some cases, heart failure [4].

## Case presentation

The patient was a 37-year-old male who had been diagnosed with MFS at a young age and confirmed by a genetic test; both his mother and brother had also been diagnosed with MFS. The patient was a former smoker. His mother was 70 years old and suffered from no cardiac diseases. There was no history of sudden deaths in the family. The patient's medical history revealed that he had experienced two episodes of pneumothorax, once in each lung, one week apart. His brother had also experienced similar episodes. The patient had no cardiac complications such as aortic rupture or mitral valve regurgitation. He did not have any other medical conditions and had not undergone any cardiac surgeries.

**Figure 1:**
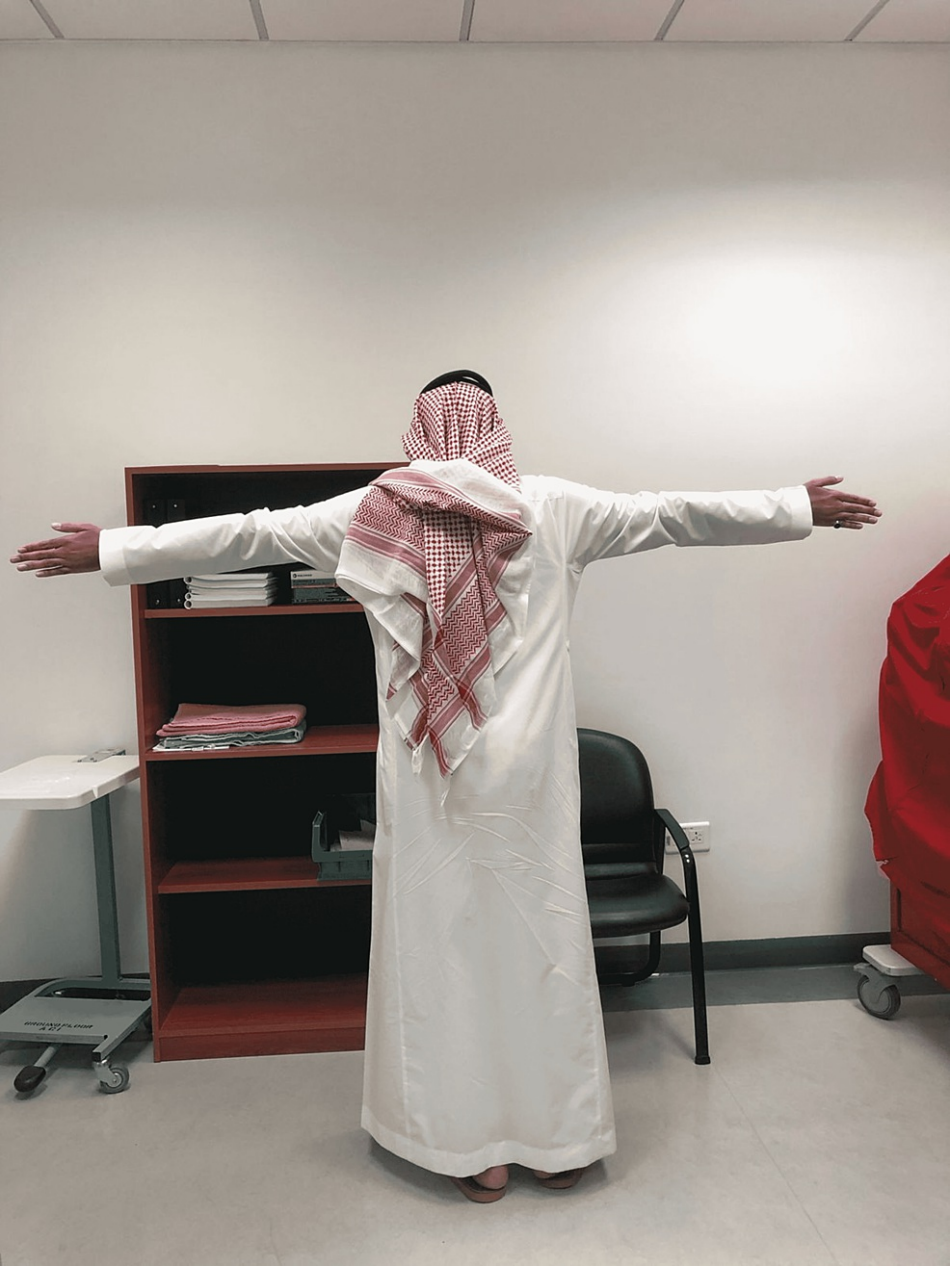
Tall stature and long arm span

**Figure 2:**
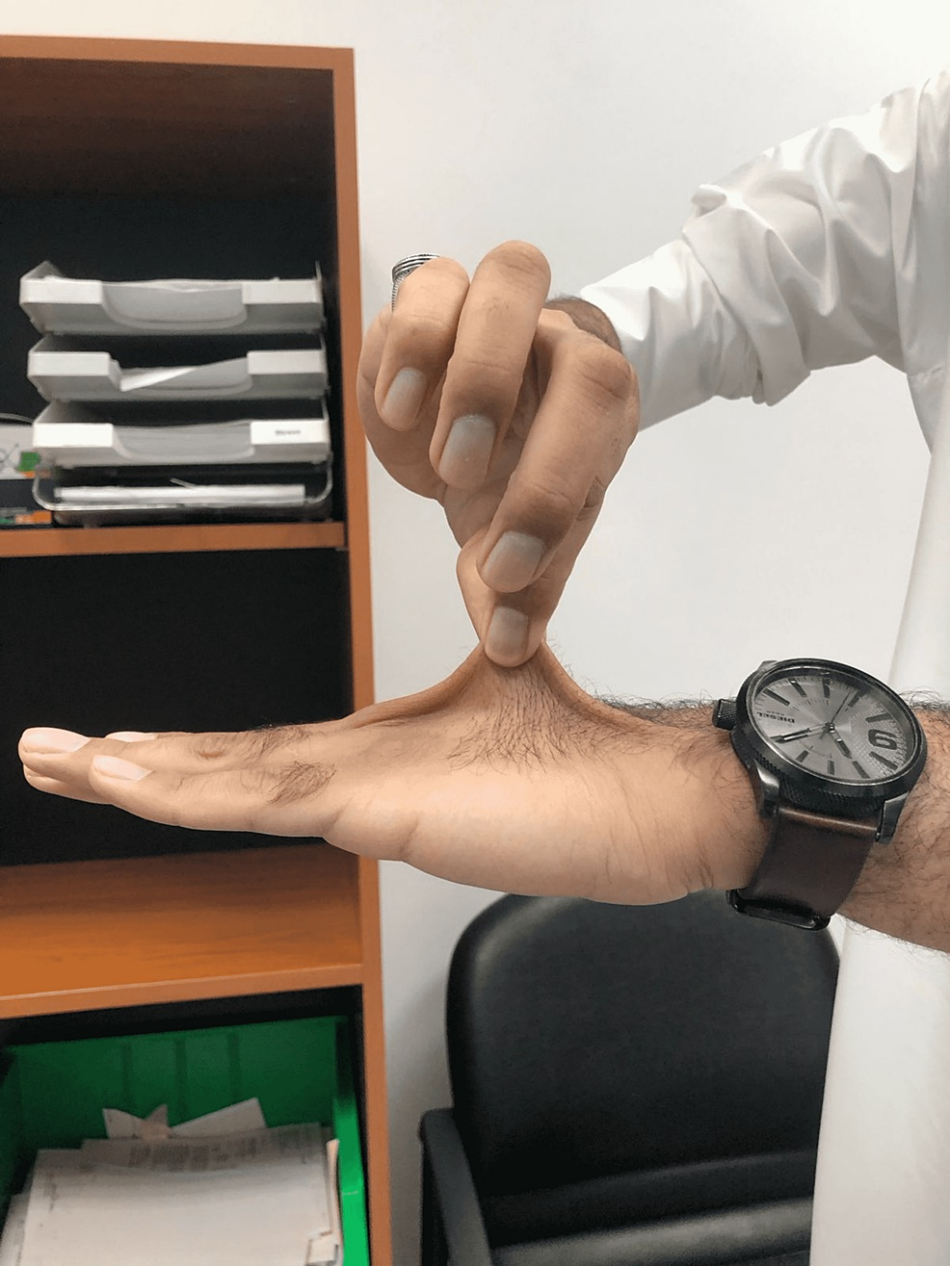
Hyper-extensive skin

On general examination, the patient had features of Marfanoid habitus including tall stature of 180 cm, long extremities, and hyper-extensive skin (Figures 1, 2). His blood pressure was normal, but the lipid profile blood test revealed high LDL levels.

**Figure 3:**
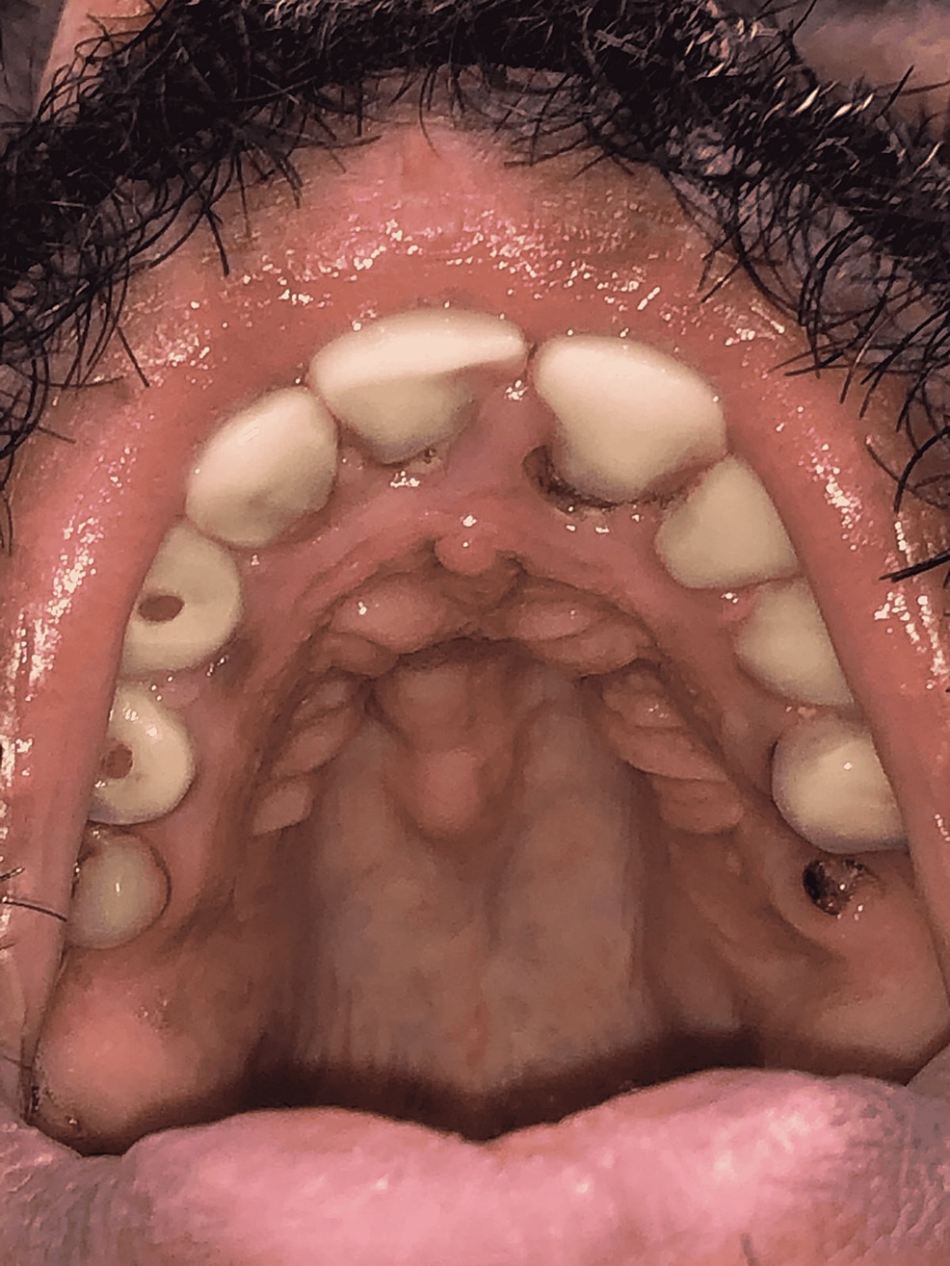
High-arched palate

There were no signs of pectus deformity, but he had mild scoliosis of the back as seen on a chest X-ray. His mouth examination revealed a high-arched palate (Figure 3).

**Figure 4:**
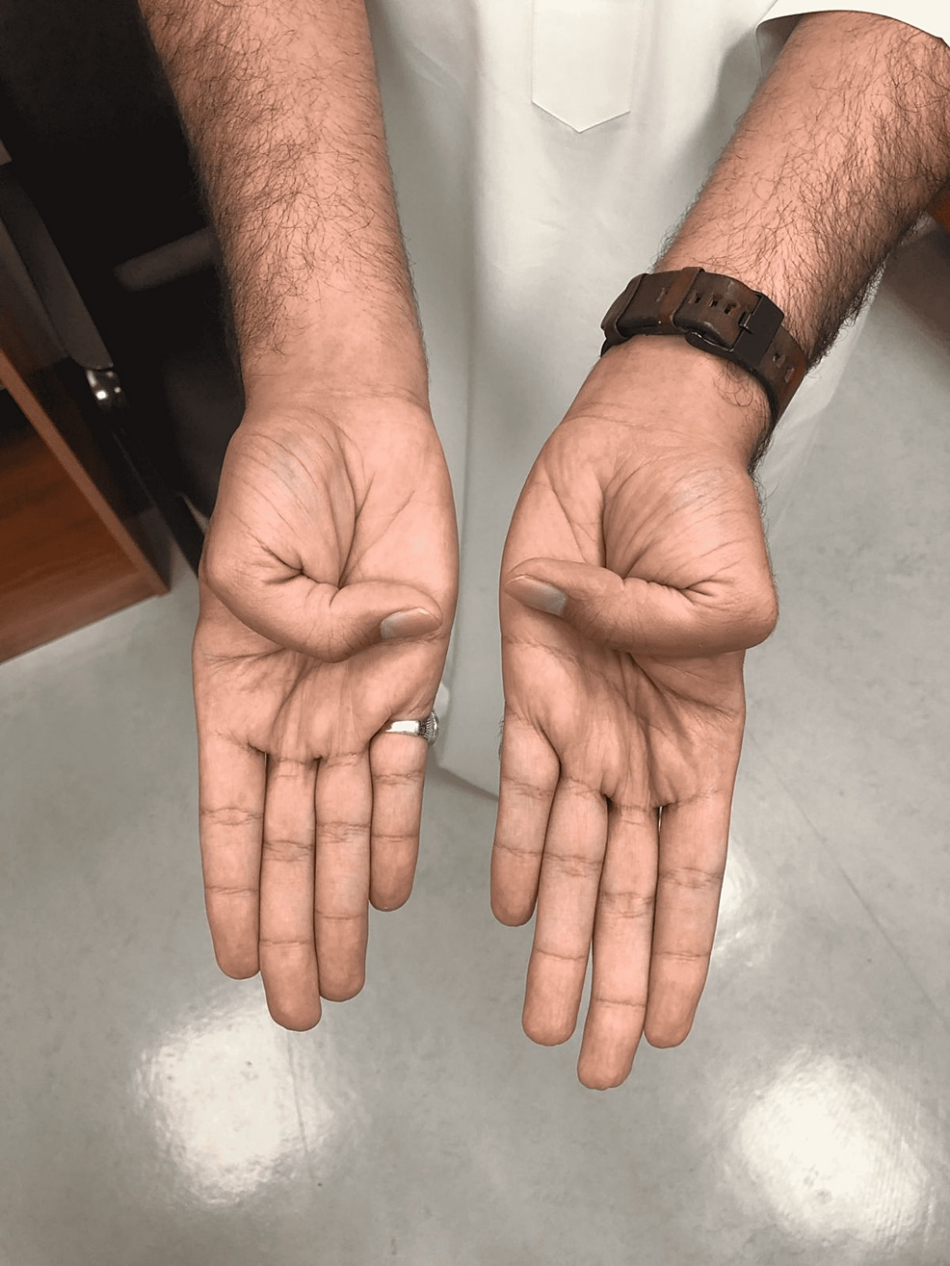
Positive thumb (Steinberg) sign

**Figure 5:**
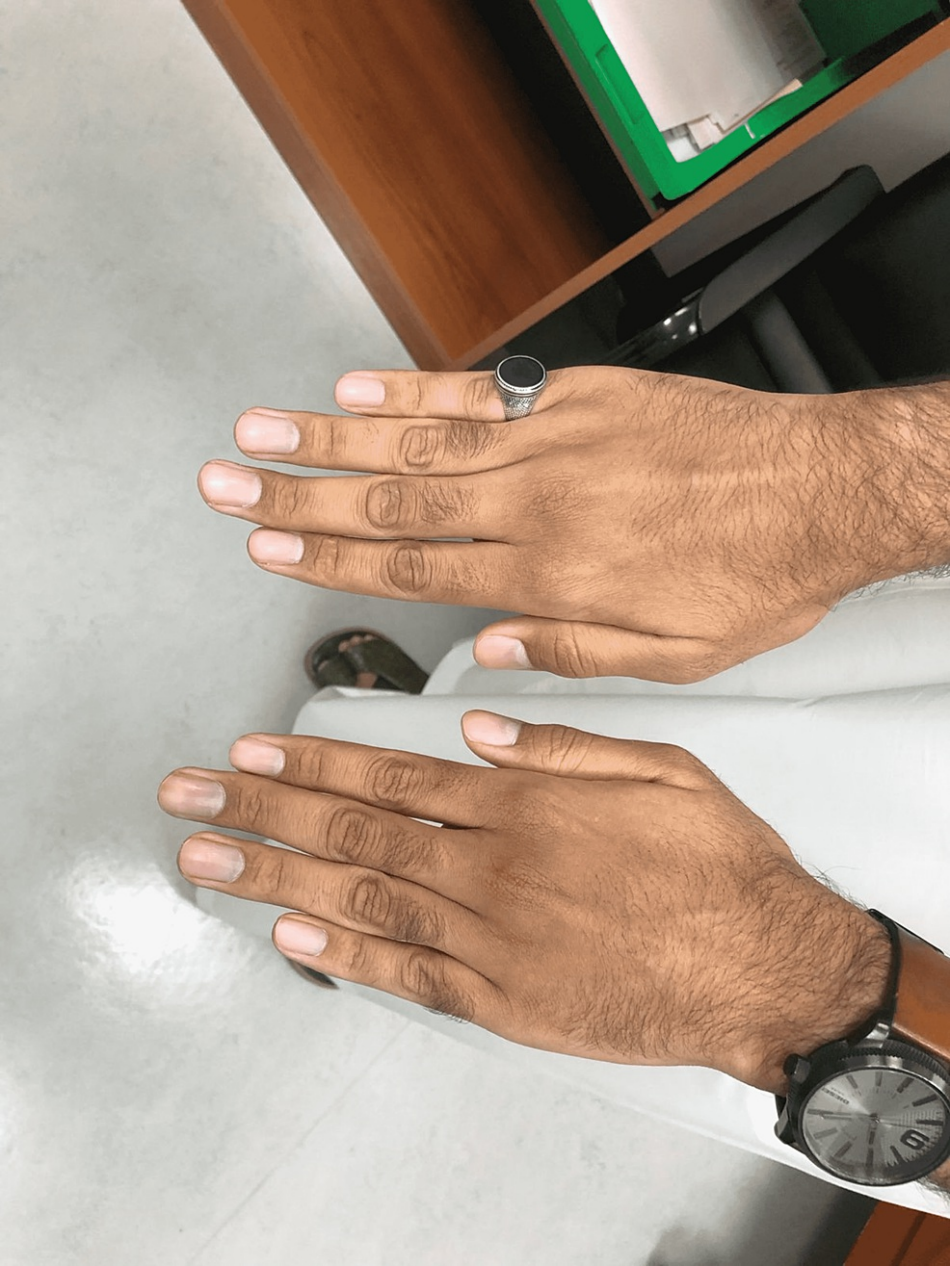
Long spider fingers (arachnodactyly)

**Figure 6:**
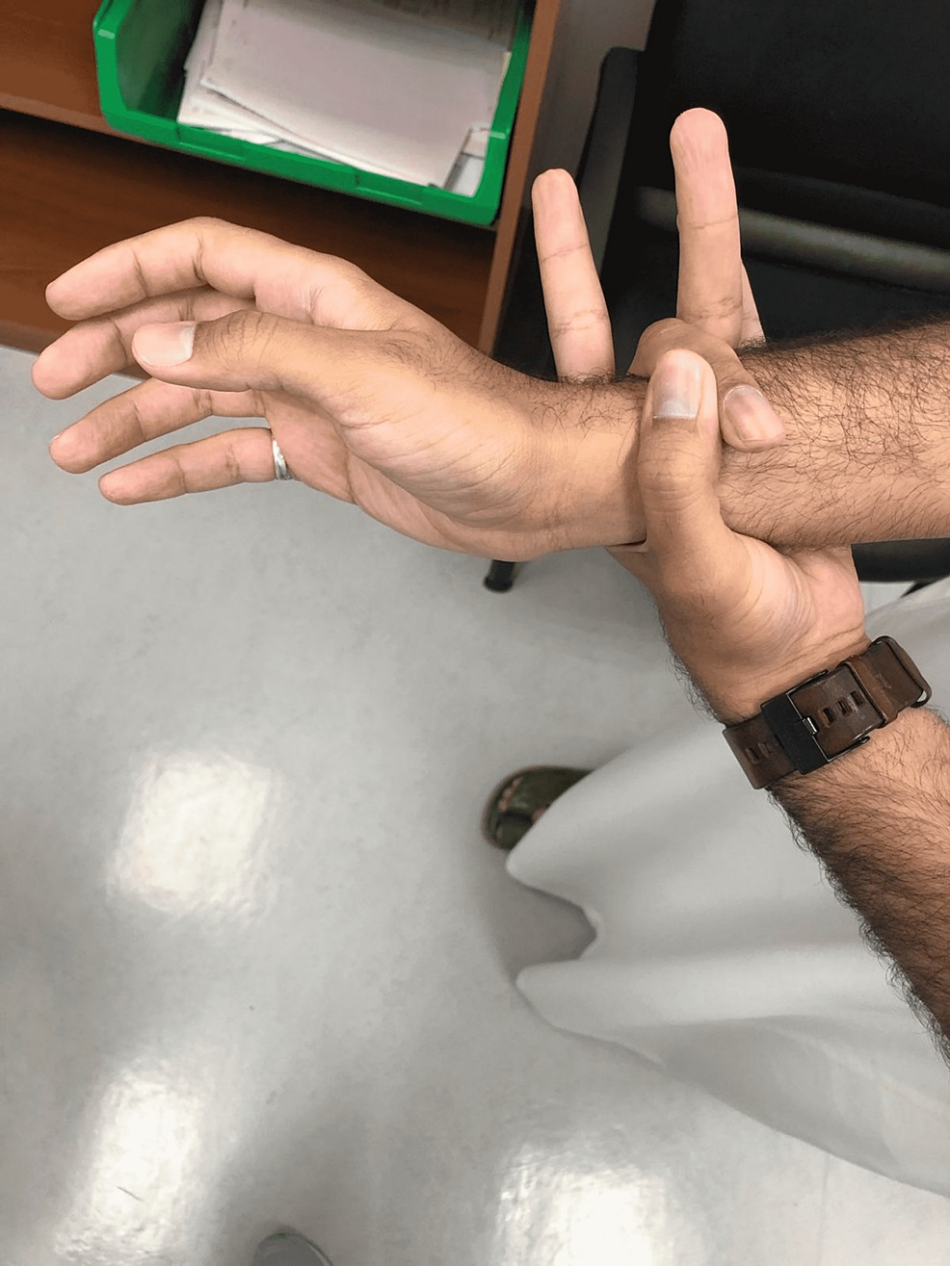
Positive wrist sign

**Figure 7:**
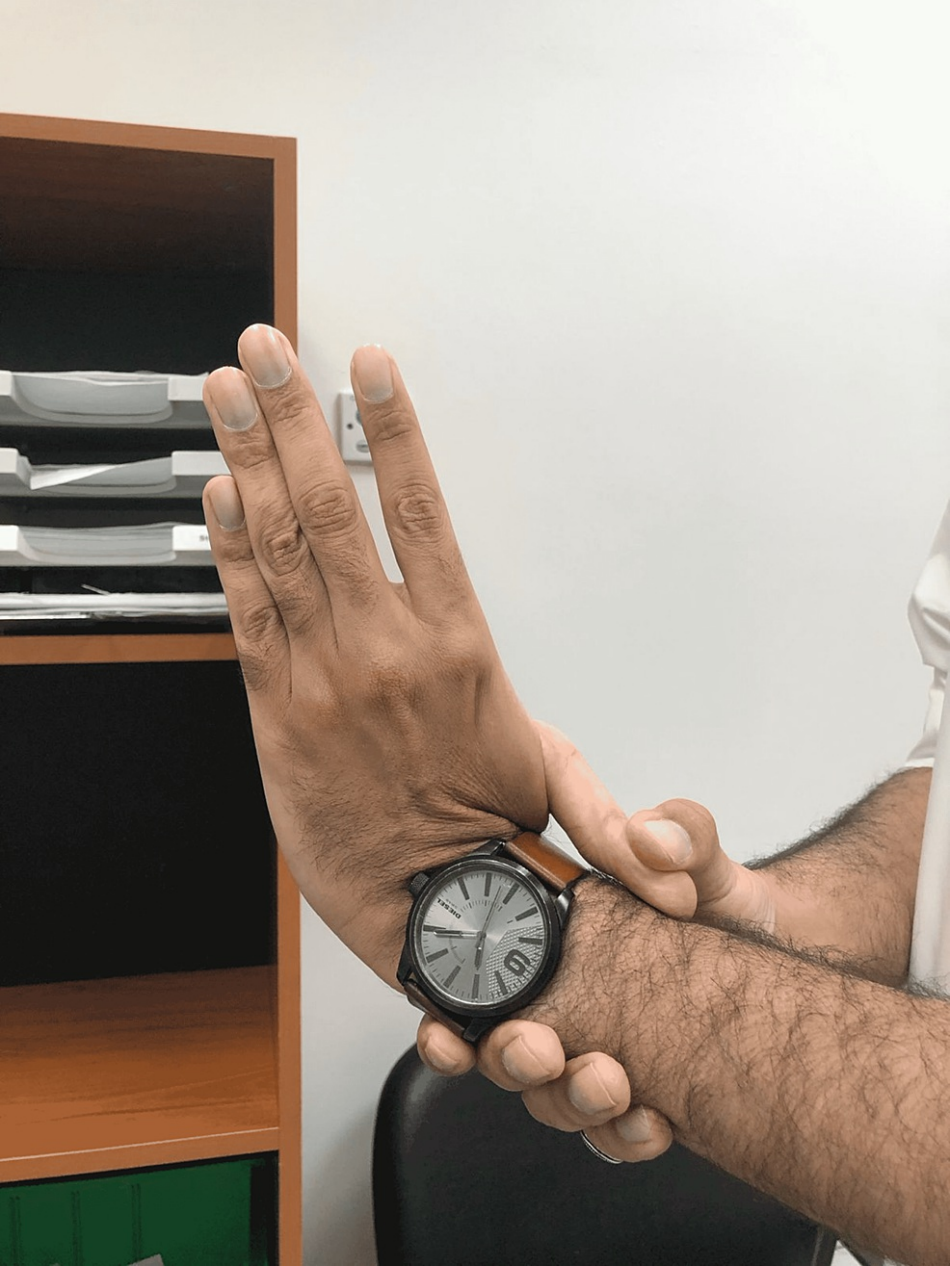
Joint hypermobility

On examination of hand signs and joint hypermobility, the patient had a positive Steinberg sign and a positive Walker-Murdoch sign, which confirmed the typical MFS hand features (Figures 4, 5, 6, 7). The patient was seen by an ophthalmologist for examination; the patient did not have ectopic lenses.

The patient underwent echocardiography, cardiac MRI, and cardiac CT in order to check for any cardiac complications (Videos 1, 2, 3, 4, 5). The echocardiography report was normal. CT showed no coronary stenosis or significant atherosclerotic plaque, and normal aorta dimension (aortic root measured 34.5 mm sinus to sinus). MRI showed normal left ventricle size with normal systolic function (LVEF=50%), normal right ventricle size and systolic function (RVEF=60%), normal tri-leaflet aortic valve, and normal aorta dimension with no aortic coarctation.

**Video 1:** Echocardiography

**Video 2:** Echocardiography 2

**Video 3:** Echocardiography 3

**Video 4:** MRI MRI: magnetic resonance imaging

**Video 5:** 3D cardiac CT of the ascending aorta CT: computed tomography

## Discussion

We presented a case of an adult male patient diagnosed with genetically confirmed MFS. The patient had an extensive family history of MFS. However, neither he nor his family had any cardiac pathology or complications.

MFS is an autosomal-dominant disorder associated with FBN1 gene mutation as well as transforming growth factor b-receptor 2 (TGFBR2) and transforming growth factor b-receptor 1 (TGFBR1) mutations [4,5]. As there is no biochemical test to confirm the diagnosis of MFS, the diagnosis is often based on a set of clinical criteria (the Ghent nosology), which includes a set of major and minor manifestations in different body systems, and it has proven to work well with the advancement in molecular techniques; confirmation of the diagnosis is possible in over 95% of patients [6].

MFS is associated with multisystemic manifestations and each system has a major criterion that should be documented to make the diagnosis: skeletal system (pectus carinatum, pectus excavatum, positive wrist and thumb sign, scoliosis >20°, reduced upper-segment to lower-segment ratio, flat foot, and protrusion acetabular) - at least four of these should be present; ocular system (lens dislocation), dura (lumbosacral dural ectasia); cardiovascular system (dilation of the ascending aorta, and dissection of the descending aorta); and the presence of family history. Additionally, the revised 2010 Ghent criteria can be used for the diagnosis depending on the absence or presence of family history.

The majority of adults with MFS have an abnormal cardiovascular system. The most common abnormalities are aortic regurgitation and aorta dilation, which occur in 60-80% of patients and can lead to aortic dissection as a complication [7]. Moreover, children with MFS are assessed for cardiac abnormalities using echocardiography, which includes a full study of the ventricles, aortic artery root dimensions, and valves.

Pulmonary manifestations could include spontaneous pneumothorax and apical blebs. In a study involving 100 patients with MFS, spontaneous pneumothorax was the most common respiratory abnormality with a prevalence of 11%. Bulla was found in five patients and bronchiectasis in two patients out of the total 100. Upper-lobe pulmonary fibrosis was diagnosed in four patients [8].

## Conclusions

MFS is an autosomal-dominant connective tissue disorder characterized by diverse clinical manifestations of the cardiovascular and musculoskeletal systems. The diagnosis of MFS is based on clinical features, but genetic testing can aid in the diagnosis. Appropriate medical and surgical treatment can lead to a good prognosis on the patient’s life expectancy.
